# The knowledge produced through student drawings

**DOI:** 10.3389/fpsyg.2022.1042383

**Published:** 2022-11-07

**Authors:** Vesife Hatisaru

**Affiliations:** ^1^School of Education, Edith Cowan University, Joondalup, WA, Australia; ^2^School of Education, University of Tasmania, Hobart, TAS, Australia

**Keywords:** draw-a-mathematician-test, draw-a-mathematics-classroom-test, legitimation code theory, student drawings, mathematics education

## Abstract

Drawings have been extensively used as a research method to gather data from research participants including school students regarding their perceptions of mathematics and its teaching and learning. What is valued in drawing-based research in mathematics education, and what kind of knowledge is produced through student drawings, however, is not known. This study examines drawing-based research studies to understand these questions by applying a novel framework – the legitimation code theory (LCT). The study focuses on two cases: one of which looked at middle school students’ images of mathematicians (draw a mathematician) and the other examined the same age group students’ descriptions of mathematics classrooms (draw a mathematics classroom). Within both studies, greater emphases are on the students’ perceptions relating to the discipline-related issues such as teaching and learning of mathematics, mathematics classroom experiences, and practices and tools of mathematicians. Students’ perceptions of the mathematics discipline and their attitudes toward mathematics and perceptions of the attributes of mathematicians are also a focus. The study offers the LCT approach to critically analyze the drawing-based research in the mathematics education field to contribute to the production of significant and needed knowledge in the field.

## Introduction

As a research method, drawings have been extensively used to collect data from school students with respect to (for instance) their views about mathematics (Rock and Shaw, 2000), mathematicians (Aguilar et al., 2016) mathematical practices (Johansson and Sumpter, 2010), their views about assessment practices in mathematics classrooms (Remesal, 2009) or high-stakes mathematics tests (Howell, 2017), and classroom practices in mathematics lessons (Pehkonen et al., 2016). A summary of the origin of the drawing-based method with a focus on mathematics education can be found in Hatisaru (2020b). Reviews of previous research using drawing-based method may be found within Hatisaru (2019a) and Hatisaru (2020a). What we know less about is what might be valued and emphasized in drawing-based research in mathematics education, and what kind of knowledge is produced through student drawings. This study aims to investigate these questions and makes an original contribution to the literature. The study follows an untypical (Niss, 2019) form that represents the variety of important elements of mathematics education research (Bakker, 2019) through a thoughtful and unique design and produces a product (Sümmermann and Rott, 2020): a way to look at drawing-based research. That is, by employing a novel framework, legitimation code theory (LCT; Maton, 2014), the study puts the spotlight on the orientations underlying to drawing-based research and offers a conceptualization that can be used to critically analyze the contribution of drawing-based research to the mathematics education field. LCT was selected as the conceptual referent for the study, as it supports analysis of knowledge practices within academic disciplines including STEM education (Winberg et al., 2019; STEM stands for science, technology, engineering, and mathematics) and perceptions of students of subject areas including mathematics, natural science, and psychology (Maton, 2007).

### Background for the study and research question

**Figure 1:**
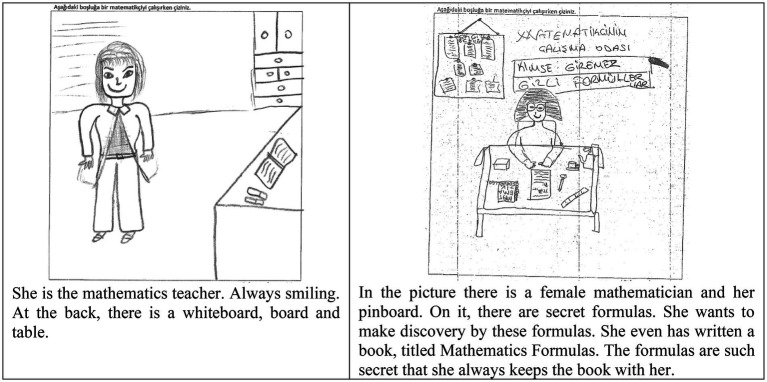
Examples of draw-a-mathematician-test (DAMT) research drawings (Hatisaru and Murphy, 2019).

**Figure 2:**
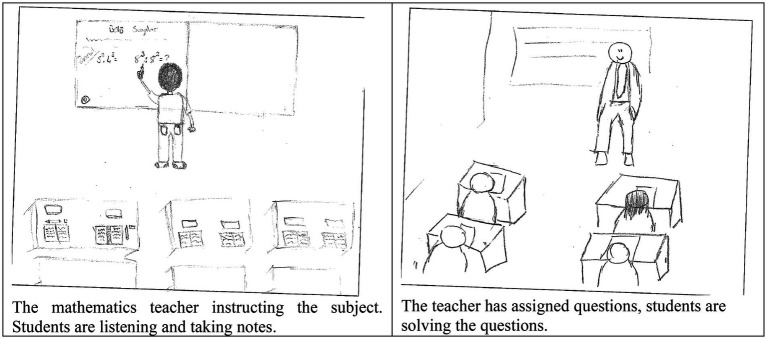
Examples of draw-a-mathematics-classroom-test (DAMC) research drawings (Hatisaru, 2020a).

The author investigated a large sample of middle school students’ perceptions of mathematicians and their work through analyzing their draw-a-mathematician-test (DAMT; Picker and Berry, 2001) pictures (hereafter referred to as the DAMT research). The students’ drawings (Figure 1) grouped into two separate categories: drawings depicting a mathematician at work (Hatisaru, 2020c), or drawings depicting a mathematics teacher in the classroom (Hatisaru, 2019a). The author explored the types of teaching in mathematics classrooms according to the students by concentrating on the latter group (Hatisaru, 2019b). This investigation showed that most students pictured a mathematics classroom where learning was predominantly directed by the teacher, and classroom practices were mainly performing procedures. However, the results were limited, as they were based on students’ drawings of mathematicians. To that end, they yielded a need for future explorations. In response to that, the author explored teaching and learning practices in mathematics classrooms by examining a sample of the same age students’ drawings of their mathematics classrooms through an adaptation of the DAMT: draw-a-mathematics-classroom-test (DAMC) (Hatisaru, 2020b; the DAMC research). The findings showed that students described mathematics classes as heavily teacher-directed where the teacher was mostly pictured at the whiteboard when lecturing, demonstrating, or explaining (Figure 2; Hatisaru, 2020a), complementing results of the previous study (Hatisaru, 2019b).

As a research method for examining students’ perceptions of mathematics classroom practices, one of the main implications of the DAMT and DAMC research studies has been that student drawings contain rich and genuine information, as also revealed in Laine et al. (2020) study. As such, the research methods used in these two research studies provide researchers with a tool to explore the codes underlying drawing-based research. The present study aims to achieve this goal. Drawing on data from the DAMT and DAMC research studies, the study investigates the question: what kind of knowledge is emphasized and produced within drawing-based research in the mathematics education field? The term ‘knowledge’ is used to indicate the new information added to the shared knowledge of the educational field through research. The term ‘codes’ is used to indicate the emphasis in a particular study or the knowledge base that is produced from it.

### An analytical framework for analyzing drawing-based research: LCT

The practice in research in general is producing knowledge. As the drawing-based methods provide an opportunity to produce knowledge in mathematics education, in this study, LCT (Maton, 2014) is used as the analytical framework. LCT is a conceptual tool used for analyzing knowledge-based practices within academic fields including online education (Maton and Chen, 2020), design disciplines (Carvalho et al., 2009), and STEM education (e.g., Winberg et al., 2019). The most elaborated dimensions of LCT are specialization, semantics, and autonomy. The core assumption of specialization is that any type of knowledge, beliefs, or practice claims are about something, and practiced by someone. Two types of relations are identified regarding specialization in a field or practice: epistemic relations (ERs) that are oriented toward an object (e.g., STEM disciplinary knowledge) and social relations (SRs) that are oriented toward a subject (e.g., STEM dispositions; Maton, 2014). Specialization (i.e., what can be objectively described as knowledge and/or who can be considered as a legitimate knower) is identified based on these relations.

The ER and SR within a specific practice, field, or event may be more strongly (+) or weakly (−) underlined in that practice, field, or event. Four main specialization codes (ER+/−, SR+/−) are originated according to their strengths (Maton and Chen, 2020). The relative strengths can be located into four quadrants in the specialization plane with infinite positions (Maton, 2014), and they form the basis of legitimation, focus or success in the relevant practice (Winberg et al., 2019). The codes that represent relative strength of relations that fit in four quadrants are: knowledge code (ER+, SR–); élite code (ER+, SR+); knower code (ER–, SR+); and relativist code (ER–, SR–).

**Figure 3:**
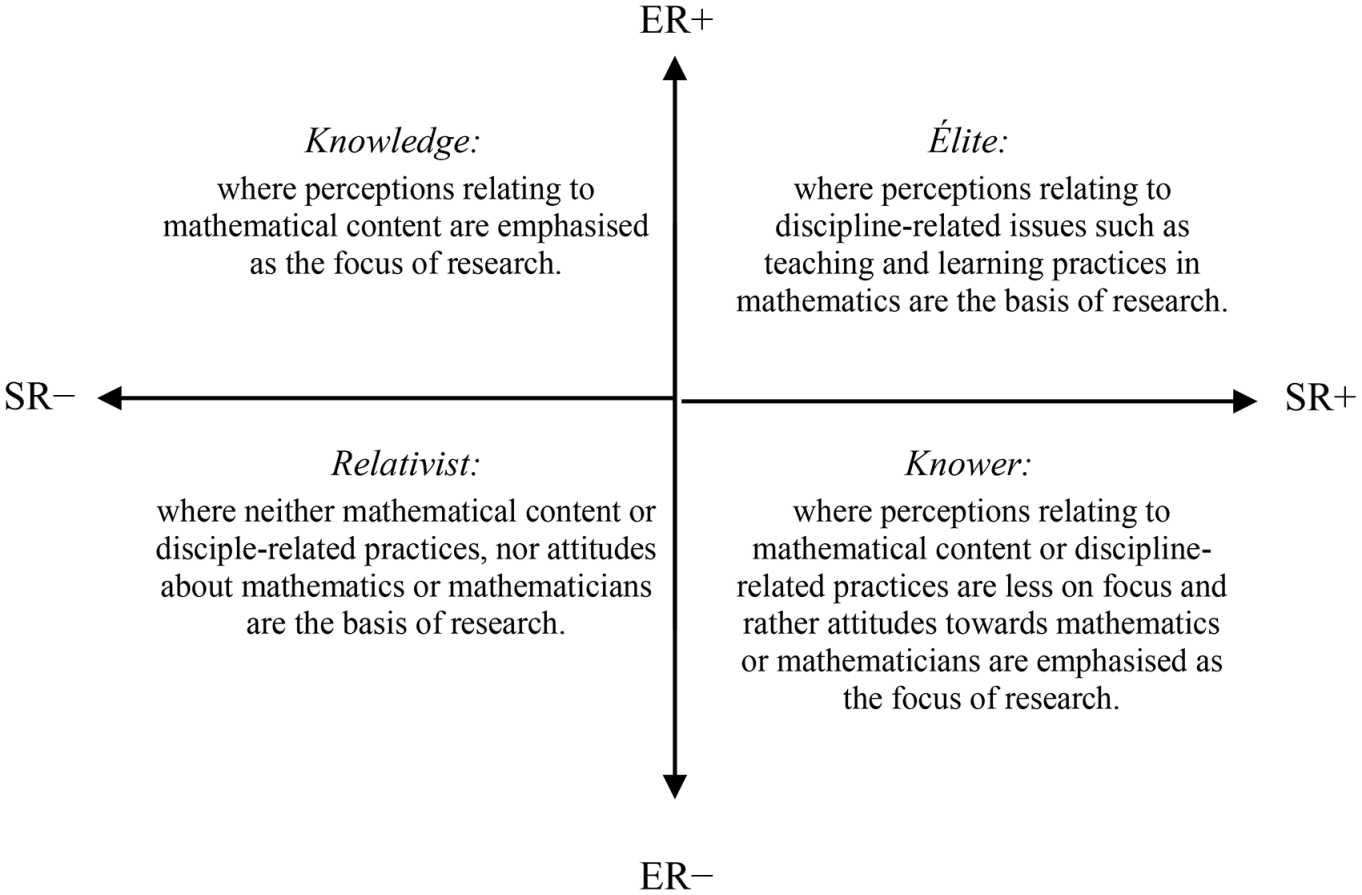
Epistemic and social relations in drawing-based research (based on Maton, 2014).

Whilst the drawing-based studies fit in the knowledge quadrant would focus on perceptions relating to the mathematical content (e.g., the concept of line, ratio, or function), studies in the élite quadrant would focus on perceptions relating to the discipline-related issues (e.g., mathematics learning experiences). Studies in the knower quadrant would focus on dispositions of individuals toward mathematics or their views about mathematicians (e.g., the mood of mathematicians), and, though it is less probable, studies in the relativist quadrant would have no/little focus on mathematical content and no/little focus on discipline-related issues (Figure 3). Therefore, these four codes provide a tool to explore the questions as follows: ‘What are the emphases in this particular drawing-based study?’ and ‘What is the knowledge base that is produced from it?’ The codes help to move beyond the surface and uncover the underpinning logic of the relevant study. Some of the drawing-based studies, for example, are likely to place much greater weight on the mathematics discipline itself, and some on the social elements of teaching and learning of mathematics, or other possibilities. By examining these codes, the underlying orientations in drawing-based research can be made more explicit.

## Materials and methods

### Study context

The current study was situated within two primarily qualitative, drawing-based research studies. The DAMT research explored middle school students’ images of mathematics in which the DAMT, (Picker and Berry, 2001), was used to generate data. The image of mathematics construct in the DAMT research comprises of students’ views about mathematicians, perceived needs for mathematics in them, and their attitudes toward mathematics (Sam and Ernest, 2000). The DAMT combined a drawing task with two open-ended written items. The drawing task included picturing a mathematician at work and next describing the picture. One open-ended item asked about possible reasons for the need for mathematics and mathematicians aiming to understand students’ perceptions of mathematics and the work of mathematicians. The other asked to complete the sentence: “To me, mathematics is …” aiming to examine students’ stated attitudes toward mathematics. Data were gathered from a total of 1,284 grades 6 to 8 students, aged 11–14, enrolled in twenty different middle schools based in Ankara, Turkey.

The DAMC research looked at the same year group students’ descriptions of mathematics classrooms. Data were collected from 400 students from three different middle schools in Ankara using the DAMC task (Hatisaru, 2020b). The students were prompted to imagine mathematics teachers and classrooms and draw a picture of their teacher teaching and themselves learning. Then they were prompted to describe the picture including activities of the depicted teacher, the students, and materials and tools that used by them. Comprehensive descriptions of the DAMT and DAMC instruments can be found in the studies presented in Supplementary Material.

The specialization plane would provide a means by which the author could investigate what is valued in the DAMT and DAMC studies, and accordingly, through student drawings, what kind of knowledge has been produced. The possible nature that ERs and SRs could reveal in these studies would vary depending on their strengths (Maton, 2014). The specialization plane would allow the focus of each study to be situated in different locations that might be viewed as reasonable, valued or more heavily weighted.

### Data analysis

A translation device is necessary in order to operationalize the analysis of the data using the specialization plane (Maton, 2014). In this study, the translation device presented in Figure 3, generated based on Maton (2014), was used for data analysis. To elaborate, ERs in the knowledge, produced through the DAMT or DAMC research studies, describe stronger or weaker perceptions relating to the mathematics disciplinary content along a continuum, from perceptions highly related to mathematical content to little or no connection. SRs in the study reveal stronger or weaker forms of perceptions relating to the teaching and learning of mathematics along a continuum, from issues highly related to the discipline-related practices in mathematics to those less related. The knowledge quadrant has stronger ERs to perceptions relating to the mathematical content and has weaker SRs to the teaching and learning of and attitudes toward mathematics (ER+, SR–), whereas the knower quadrant has weaker ERs to perceptions relating to the mathematical content or discipline-related practices, and instead has stronger relations to attitudes toward mathematics (ER–, SR+). The élite quadrant has stronger relations to discipline-related issues (ER+, SR+).

The focus of each study, their research aim/questions and data analysis aspects, was analyzed using the lens of the specialization plane. Where the focus of a particular study foregrounded students’ perceptions relating to the mathematics discipline (e.g., types of mathematical tasks), this aspect was interpreted as displaying a predominantly knowledge code. In contrast, where the focus of a study demonstrated aspects relating to students’ attitudes toward mathematics or their views about mathematicians (e.g., mood of the mathematics teacher), it was clear that some knowledge on the views about mathematicians was produced, demonstrating a knower code. Where the focus was mainly on discipline-related practices of mathematics or mathematicians (e.g., the teacher’s classroom activity), this was interpreted as demonstrating an élite code. A particular study might demonstrate more than one focus, and more than one code accordingly.

**Figure 4:**
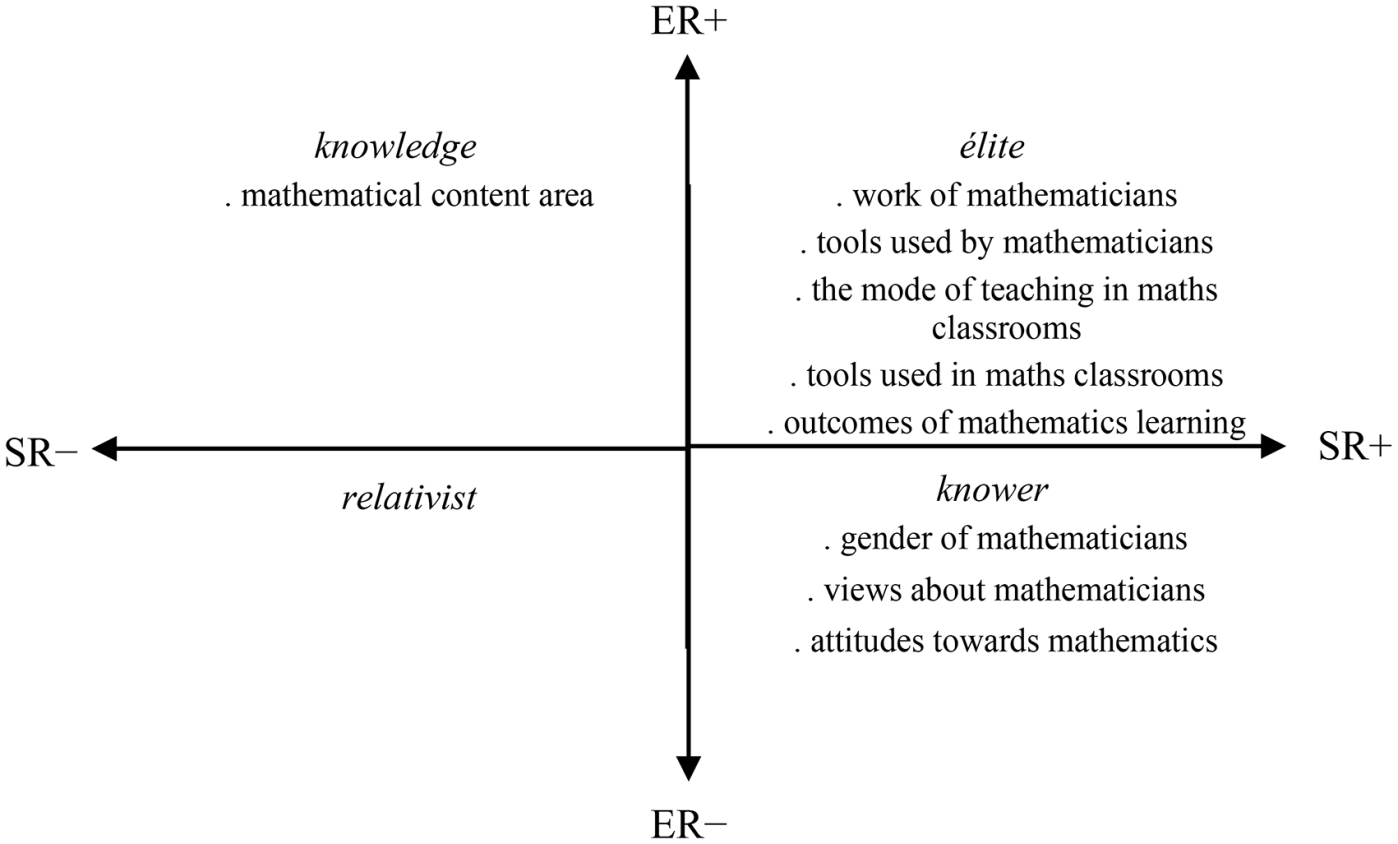
Mapping of the DAMT study focuses on the specialization plane.

**Figure 5:**
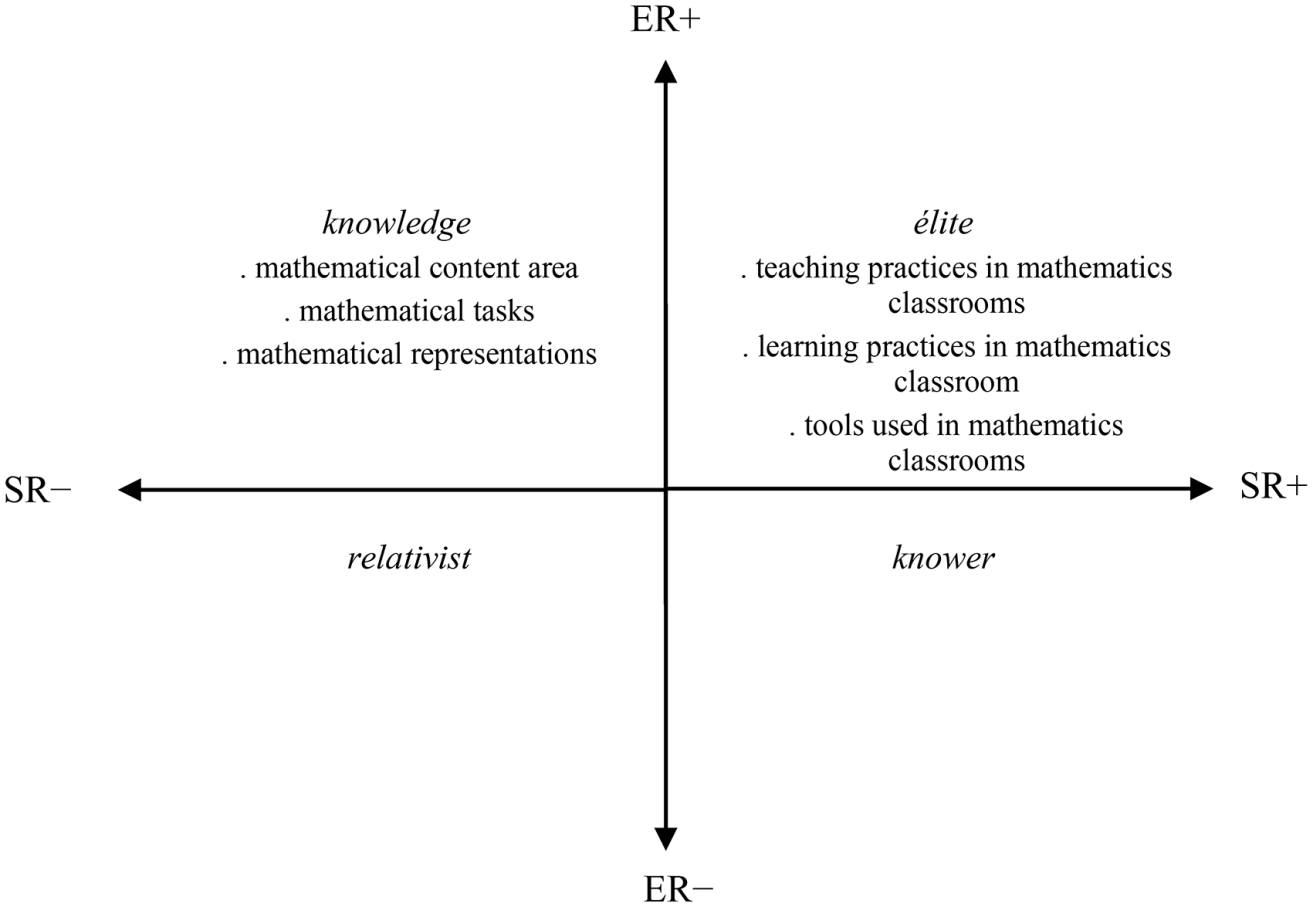
Mapping of the DAMC study focuses on the specialization plane.

The analysis was intended to capture the general gist of the practice in the DAMT and DAMC studies, as opposed to a fine-grained micro-analysis, and this approach is defined as soft focus in LCT (Maton, 2014). This soft-focus analysis was applied to each of the studies. The analysis process then shifted to visualizing the focuses of each study on the specialization plane and positioning them on it (Figures 4, 5). These analyzes are presented and elaborated in the following section.

## Findings

### The DAMT research

The focuses of the DAMT studies are located predominantly in the élite and knower quadrants, and to a lesser extent in the knowledge quadrant (Figure 4). As elaborated earlier, the knowledge code represents a study focus which foregrounds ERs and backgrounds SRs (ER+, SR–). Within these studies, the knowledge produced representing the knowledge code includes students’ perceptions of the mathematical content area. It is reported that Algebra, Numbers and Operations, and Geometry were the remarkable mathematical content domains in the students’ DAMT portrayals (e.g., Hatisaru, 2019a, 2020c). In addition to these, some mathematical theorems (e.g., Hasse-Arf Theorem) were captured in a few students’ drawings which depicted a mathematician (Hatisaru, 2020c).

The élite code foregrounds both epistemic and SRs (ER+, SR+). The knowledge produced representing this code is typically where students’ perceptions relating to the work of, and the tools used by, mathematicians (Hatisaru, 2020c) and mathematics teachers (Hatisaru, 2019a) are provided. The findings include that, according to the students, the chief discipline-related activities of mathematicians were studying or creating mathematics, and the chief activities of mathematics teachers were teaching, explaining, or demonstrating. The primary tools used by mathematicians and mathematics teachers included a whiteboard and books, and in a few cases concrete materials and technological tools (Hatisaru, 2019a, 2020c).

Students perceived their mathematics classrooms to be chiefly teacher-directed where the teacher was at the center of learning. There was little group or peer work, and the main classroom resources were a whiteboard and books. In a few drawings where the mode of instruction identified was potentially more student-oriented, students were found to be happier than in drawings where the mode of instruction was chiefly teacher-directed (Hatisaru, 2019b). Mathematics was found useful or necessary for basic everyday life tasks by most of the students such as performing financial calculations or using arithmetic, while some students found it useful for doing university studies. A few of the students thought that mathematics was necessary for problem solving, and a few others viewed mathematics as underpinning science and technology (Hatisaru, 2020d).

The knower code represents a study focus which foregrounds SRs and backgrounds ERs (ER–, SR+). The students’ perceptions of the gender and attractiveness of mathematicians and mathematics teachers are among the knowledge produced representing this code (Hatisaru, 2019a, 2020c). It is reported that the students exhibited occupational gender stereotypes. They mostly viewed mathematics teachers as female (Hatisaru, 2019a) and mathematicians as male (Hatisaru, 2020c). While the female teacher stereotype became less strong by age group (i.e., fewer grade 8 students depicted the teacher as female compared to 6th and 7th-graders) (Hatisaru, 2019a), the male stereotype did not change. Many of the students at each grade level pictured male mathematicians (Hatisaru, 2020c). In general, the students reflected a positive mathematics teacher image which was smiley or serious and dedicated, while a small group of students expressed a relatively negative image of mathematics teachers which was angry or silly (Hatisaru, 2019a). Like mathematics teachers, many of the students associated positive feelings with mathematicians depicting smiley or serious, focused and dedicated mathematicians. Only a small number of students pictured a mad, angry, or silly mathematician (Hatisaru, 2020c).

The students’ stated attitudes toward mathematics were found to be generally positive, whereas a small percentage of them stated negative feelings. Most of the responses to “what mathematics means to me” were in the nature mathematics is “an enjoyable subject,” “very important” and/or “necessary” (Hatisaru and Murphy, 2019). An interesting observation was that the perceived negative image of the mathematics teacher could result in feeling unhappy in mathematics classrooms or a loathing of mathematics (Hatisaru, 2019a). Within a further investigation taken into how students’ stated attitudes toward mathematics are influenced by their perceptions of the teacher, it was suggested that some of the students who perceive their teacher a ‘creature’ still might associate positive feelings with mathematics, as they find mathematics important and necessary for schooling, but some of them might dislike mathematics or have mixed feelings with respect to the need for learning mathematics due to their negative perceptions of the teacher (Hatisaru and Murphy, 2019).

### The DAMC research

The focuses of DAMC studies are located in the knowledge and élite quadrants (Figure 5). As discussed earlier, the knowledge code represents a study focus which emphasizes perceptions relating to the specialized knowledge of mathematics discipline. The study focuses representing the knowledge code include students’ perceptions of the mathematical content area, and the types of mathematical tasks used in mathematics classrooms and their representational form (symbolic, visual, verbal) (Hatisaru, 2020b). Findings revealed that mathematics was predominantly represented through symbolic representations in the students’ drawings. Contextual or real-life based and open-ended tasks were not common, while the tasks that focused on procedural skills were the most usually included. Symbolic representations dominated student responses where the mathematical tasks were most commonly represented through numbers, equations, and expressions. Only a few of the students used verbal, visual or graphical representations (Hatisaru, 2020b).

The élite code emphasizes perceptions relating to how mathematics is taught or learned. The students’ perceptions of the teaching and learning practices in their mathematics classroom, and materials and resources used in the teaching and learning of mathematics, (Hatisaru, 2020a) represent this code. It was found that the students perceived their mathematics classroom as chiefly teacher directed. That is, the teacher is the conductor of learning and instruction. The teacher usually demonstrates and explains the content and asks or solves closed mathematics questions with one answer (e.g., 2*x* − 3 = 7, find *x*). Students are relatively passive; they listen to the teacher who is at the center of class and teaches. The class usually engages in solving routine questions. There are almost no content-related interactions among students in the classroom, and interactions between the teacher and students are limited to the teacher asking routine mathematics questions and students giving responses to them. The main teaching and learning resources are a whiteboard and notebooks or textbooks (Hatisaru, 2020a).

## Discussion and conclusion

**Figure 6:**
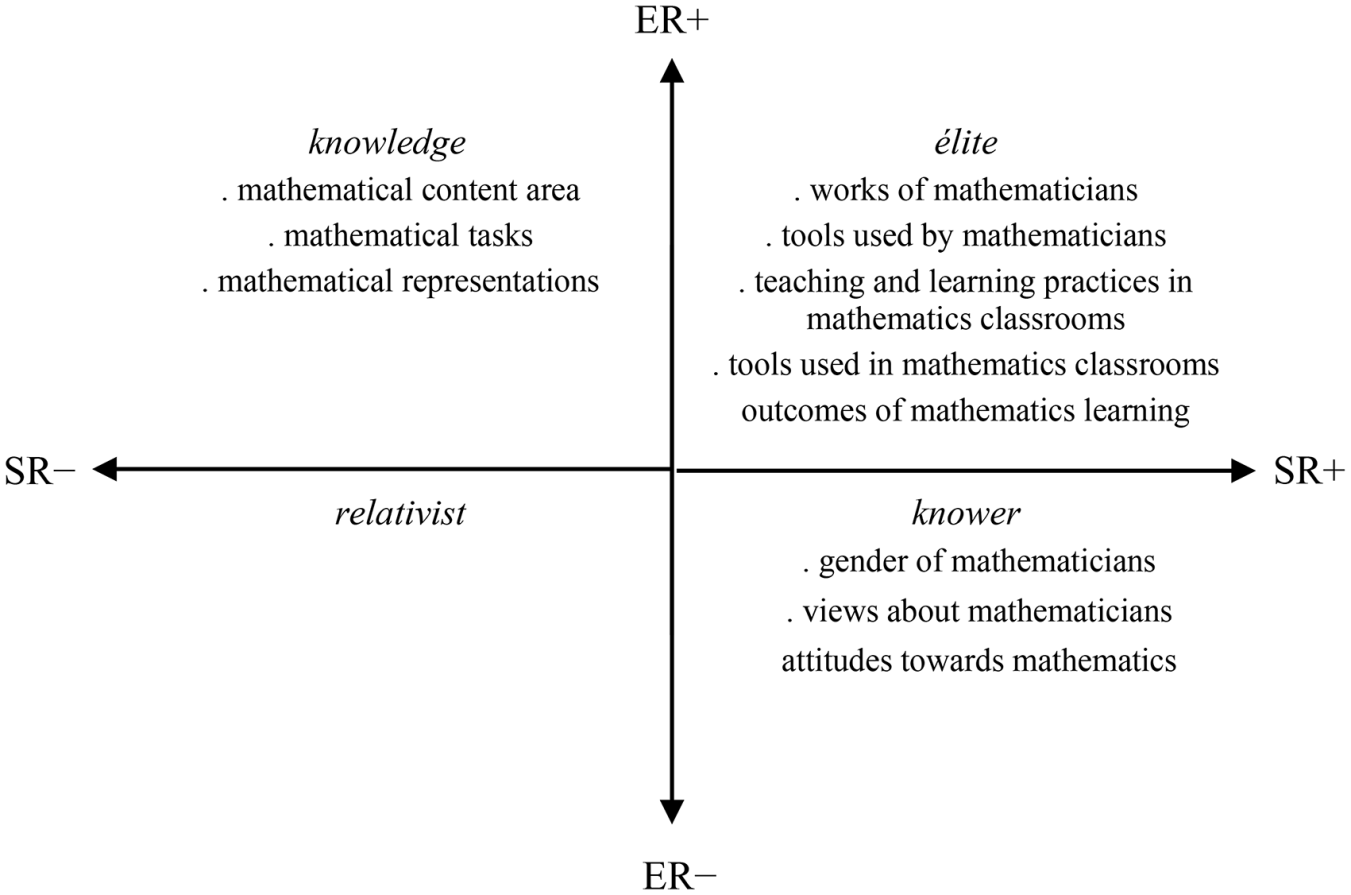
Mapping of the DAMT and DAMC study focuses on the specialization plane.

The focuses or emphasis (dominant codes) within drawing-based research practices are not generally discussed in mathematics education literature. In this study, what might be valued and emphasized on within drawing-based research is explored by applying the LCT (Maton, 2014) to two drawing-based research studies. They are the DAMT which investigated middle school students’ images of mathematicians (Hatisaru and Murphy, 2019; Hatisaru, 2019a, 2019b, 2020c, 2020d) and the DAMC which examined the same age group students’ descriptions of mathematics classrooms (Hatisaru, 2020a, 2020b) studies (Supplementary Material). The focuses of the two cases are distributed over three quadrants in the LCT specialization plane with a significant involvement in the élite quadrant. This indicates that, within both cases, greater emphases were on the students’ perceptions relating to the discipline-related issues such as teaching and learning of mathematics, mathematics classroom experiences, and practices and tools of mathematicians. Students’ perceptions of the mathematics discipline and their attitudes toward mathematics and perceptions of the attributes of mathematicians were also a focus, located in the knowledge and knower quadrants, respectively (Figure 6).

The study has neither intended to review all existing drawing-based research studies in the field nor has suggested that knowledge-code studies are significant than knower-code studies, or vice versa. As Maton (2014) indicates, there are many contexts within which knower-code studies are needed and many others within which knowledge-code, or élite-code, studies. Rather, drawing data from two cases, the study aimed to illustrate how LCT offers an approach to investigate the kinds of knowledge produced through student drawings. Employing this novel approach, fruitful insights may arise about what drawing-based research studies within the mathematics education field put greater emphasis on. Using the specialization plane not only reveals what is produced, but also shows the gaps in the literature. That is, whether more research is needed to address students’ perceptions of mathematical ideas, concepts, and procedures (knowledge-code studies), or key issues relating to the teaching and learning of mathematics (élite-code studies), or students’ dispositions about mathematics or mathematicians (knower-code studies). To that end, this approach potentially contributes to the production of significant and needed knowledge in the field, and this is where further investigations are warranted.

## Limitations, future directions

This study investigated the question: What kind of knowledge is emphasized and produced within drawing-based research in the mathematics education field? While the findings provide very useful information about the potential emphasis in drawing-based research studies, and the new knowledge added to the shared knowledge of the educational field through them, there are three limitations of the study that need to be considered. First, to the author’s knowledge, the LCT has not been used yet for investigating knowledge practices in drawing-based research in the mathematics education field, whilst it has been widely used for researching knowledge practices based on the other forms of data (e.g., textual; e.g., Maton, 2007; Winberg et al., 2019). As one of the first attempts, the author has generated the translation device presented in Figure 3 based on the existing research. Other mathematics education researchers may generate different translation devices and plausibly may find different or additional findings to those found in this study. The author hopes that mathematics education researchers will pursue this possibility.

Second, examination of all relevant drawing-based research studies was not the intention of the study; rather, the study focused on two cases. Results may vary depending on the context of drawing-based research studies that are examined and the sample size. The study, however, offers a conceptualization that can be used to critically analyze the contribution of drawing-based research in the mathematics education field. Follow-up studies are recommended using the operationalizations developed in this study on the existing drawing-based research studies in mathematics education. More importantly, the study approach may provide researchers with useful insight regarding identifying the gaps in the literature: i.e., whether more research is needed to address students’ perceptions of mathematical ideas, concepts, and procedures (knowledge-code studies), or key issues relating to the teaching and learning of mathematics (élite-code studies), or students’ dispositions about mathematics or mathematicians (knower-code studies).

Third, the data was analyzed by the author, who had conducted the DAMT and DAMC research studies. Relying on pre-existing self-reported data might have weakened the validity of data analysis, and the author employed several validation processes to overcome that limitation. For example, the translation device employed in this study (Figure 3) was generated based on Maton (2014) and the author’s earlier applications of the LCT to STEM education (e.g., Hatisaru, 2021). Those earlier LCT works were helpful to refine definitions of the codes in this study before applying them to the two cases. Moreover, findings from the two cases were presented comprehensively in the Findings section. These rich descriptions not only contribute to the validity check mechanism but are also useful for understanding students’ perceptions of mathematics, mathematicians, teaching and learning practices in mathematics classrooms. Finally, mapping of the focuses and data analysis aspects in the DAMT and DAMC studies with the four LCT codes was presented in Supplementary Material to give the reader a sense of how LCT codes were used in data analysis.

## Data availability statement

The original contributions presented in the study are included in the article/Supplementary material, further inquiries can be directed to the corresponding author.

## Author contributions

The author confirms being the sole contributor of this work and has approved it for publication.

## Conflict of interest

The author declares that the research was conducted in the absence of any commercial or financial relationships that could be construed as a potential conflict of interest.

## Publisher’s note

All claims expressed in this article are solely those of the authors and do not necessarily represent those of their affiliated organizations, or those of the publisher, the editors and the reviewers. Any product that may be evaluated in this article, or claim that may be made by its manufacturer, is not guaranteed or endorsed by the publisher.

## References

[ref1] Aguilar M. S. Rosas A. Zavaleta J. Romo-Vázquez A. (2016). Exploring high-achieving students' images of mathematicians. Int. J. Sci. Math. Educ. 14, 527–548. doi: 10.1007/s10763-014-9586-1

[ref2] Bakker A. (2019). What is worth publishing? A response to Niss. Learn. Math. 39, 43–45.

[ref3] Carvalho L. Dong A. Maton K. (2009). Legitimating design: a sociology of knowledge account of the field. Des. Stud. 30, 483–502. doi: 10.1016/j.destud.2008.11.005

[ref5] Hatisaru V. (2019a). Lower secondary students’ views about mathematicians depicted as mathematics teachers. LUMAT 7, 27–49.

[ref6] Hatisaru V. (2019b). “Putting the spotlight on mathematics classrooms,” in Proceedings of the International Symposium Elementary Mathematics Teaching (SEMT). eds. Novotná J. Moraová H., 182–192.

[ref7] Hatisaru V. (2020a). School students’ depictions of mathematics teaching and learning practices. Int. Electron. J. Elementary Educ. 13, 199–214.

[ref8] Hatisaru V. (2020b). Exploring evidence of mathematical tasks and representations in the drawings of middle school students. Int. Electron. J. Math. Educ. 15, 1–21. doi: 10.29333/iejme/8482

[ref9] Hatisaru V. (2020c). “[He] has impaired vision due to overworking”: students’ views about mathematicians,” in Theorizing and Measuring Affect in Mathematics Teaching and Learning. eds. Andrà C. Brunetto D. Martignone F. (Cham: Springer), 89–100.

[ref10] Hatisaru V. (2020d). Perceived need for mathematics among lower secondary students. Aust. Math. Educ. J. 2, 9–14.

[ref11] Hatisaru V. (2021). “The views of STEM specialisation among STEM educators,” in British Society for Research into Learning Mathematics (BSRLM) Proceedings. ed. Marks R., *Vol*. 41.

[ref12] Hatisaru V. Murphy C. (2019). Creature' teachers 'Monster' mathematicians: Students' views about mathematicians and their stated attitudes to mathematics. Int. J. Educ. Math. Sci. Technol. 7, 215–221.

[ref13] Howell A. (2017). ‘Because then you could never ever get a job!’: children’s constructions of NAPLAN as high-stakes. J. Educ. Policy 32, 564–587. doi: 10.1080/02680939.2017.1305451

[ref14] Johansson D. A. Sumpter L. (2010). “Children’s conceptions about mathematics and mathematics education,” in Proceedings of the MAVI-16 Conference. ed. Kislenko K. (Tallinn University of Applied Sciences), 77–88.

[ref15] Laine A. Ahtee M. Näveri L. (2020). Impact of teachers’ actions on emotional atmosphere in mathematics lessons in primary school. Int. J. Sci. Math. Educ. 18, 163–181. doi: 10.1007/s10763-018-09948-x

[ref16] Maton K. (2007). “Knowledge–knower structures in intellectual and educational fields,” in Language, Knowledge and Pedagogy. eds. Christie F. Martin J. R. (Continuum), 87–108.

[ref17] Maton K. (2014). Knowledge and Knowers: Towards a Realist Sociology of Education Routledge.

[ref18] Maton K. Chen R. T.-H. (2020). “Specialization codes: knowledge, knowers and student success,” in Accessing Academic Discourse: Systemic Functional Linguistics and Legitimation Code Theory. eds. Martin J. R. Maton K. Doran Y. J. (Routledge), 35–58.

[ref19] Niss M. (2019). The very multi-faceted nature of mathematics education research. Learn. Math. 39, 2–7.

[ref20] Pehkonen E. Ahtee M. Laine A. (2016). “Pupils’ drawings as a research tool in mathematical problem-solving lessons,” in Posing and Solving Mathematical Problems: Advances and New Perspectives (Research in Mathematics Education). eds. Felmer P. Pehkonen E. Kilpatrick J. (Springer), 167–188.

[ref21] Picker S. Berry J. (2001). Your students’ images of mathematicians and mathematics. Math. Teach. Middle Sch. 7, 202–208. doi: 10.5951/MTMS.7.4.0202

[ref22] Remesal A. (2009). “Accessing primary pupils’ conceptions of daily classroom assessment practices,” in Students’ Perspectives on Assessment: What Students Can Tell us About Assessment for Learning. eds. McInerney D. M. Brown G. T. L. Liem G. A. D. (Information age Publishing, Inc), 25–51.

[ref23] Rock D. Shaw J. M. (2000). Exploring children’s thinking about mathematicians and their work. Teach. Child. Math. 6, 550–555. doi: 10.5951/TCM.6.9.0550

[ref24] Sam L. C. Ernest P. (2000). A survey of public images of mathematics. Res. Math. Educ. 2, 193–206. doi: 10.1080/14794800008520076

[ref25] Sümmermann M. L. Rott B. (2020). On the future of design in mathematics education research. Learn. Math. 40, 31–34.

[ref26] Winberg C. Adendorff H. Bozalek V. Conana H. Pallitt N. Wolff K.. (2019). Learning to teach STEM disciplines in higher education: a critical review of the literature. Teach. High. Educ. 24, 930–947. doi: 10.1080/13562517.2018.1517735

