# Supplementary material for: The knowledge produced through student drawings
Source: Front Psychol. 2022 Nov 7;13:1042383. doi: 10.3389/fpsyg.2022.1042383 (PMC9676250; doi:10.3389/fpsyg.2022.1042383)
Supplement: Supplementary file 1 [file Table_1.docx]

**Study cases and their focuses**

| **The case** | **Research study** | **The focus**  **research aim/questions** | **data analysis aspects** | **sub-aspects** |
| --- | --- | --- | --- | --- |
| Draw a Mathematician research (DAMT) | Hatisaru (2019a) | What views do lower secondary students have about mathematicians when depicted as a mathematics teacher? | Gender *(knower code)*; Physical environment *(knower code)*; Activity *(élite code)*; Mathematical content area *(knowledge code)*; The tools of the profession *(élite code)*; Attractiveness of mathematics teachers *(knower code)*. | Female, male; Classroom, office; Teaching, working, researching, reading; Algebra, numbers and operations, geometry; Whiteboard, books, concrete materials, exam papers, pinboard, technological tools; Smiling, serious, thinking, angry, silly, bored. |
|  | Hatisaru (2020c) | What views do students have about mathematicians and their work? | Gender *(knower code)*; Physical environment *(knower code)*; Activity *(élite code)*; Mathematical content area *(knowledge code)*; The tools of the profession *(élite code)*; Attractiveness of mathematicians *(knower code)*. | Female, male; Office, classroom, outdoor, library; Studying maths, teaching, creating maths, in the field; Algebra, numbers and operations, geometry, Hasse-Arf theorem; Whiteboard, books, concrete materials, pinboard, technological tools; Smiley, serious, focused, dedicated, mad, angry, silly. |
|  | Hatisaru and Murphy (2019) | What are the connections between students’ views about mathematicians and their stated attitudes to mathematics? | Attitudes towards mathematics *(knower code)*; Attractiveness of mathematicians *(knower code)*. | Positive, somewhat positive, mixed, somewhat negative, negative, neutral; ‘creature’ teacher, ‘monster’ mathematician. |

**(Study cases and their focuses – continued)**

| **The case** | **Research study** | **The focus**  **research aim/questions** | **data analysis aspects** | **sub-aspects** |
| --- | --- | --- | --- | --- |
| Draw a Mathematician research (DAMT) | Hatisaru (2019b) | Through the students’ eyes, in mathematics classrooms: (1) What are the modes of instruction? and (2) What resources are used? | The mode of teaching *(élite code)*; Tools *(élite code)*. | Highly student-centred, moderately student-centred, moderately teacher-directed, highly teacher-directed; Whiteboard, books, smart board, concrete materials (ruler, compass, protractor). |
|  | Hatisaru (2020d) | What views do students have about the needs for mathematics? | Outcomes of mathematics education for students *(élite code)* | Functional numeracy, work-related knowledge, specialist knowledge, images about mathematics, mathematical confidence, mathematical problem solving, critical citizenship, appreciation of mathematics (Ernest, 2015). |
| Draw a Mathematics Classroom research (DAMC) | Hatisaru (2020a) | Through the students’ eyes, in their mathematics classroom: What are the teaching practices of a teacher? What are the learning practices of students? What materials and tools are used? | Teaching activity *(élite code)*; Learning activity *(élite code)*; Materials and tools used *(élite code)*. | Disciplining, instructing, solving/asking questions; Watching or listening, responding or solving questions, working in groups or in pairs; Whiteboard, textbooks, digital tools, calculators. |
|  | Hatisaru (2020b) | What is the nature of mathematical tasks in students’ drawings? What forms of mathematical representations are used? | Mathematical tasks *(knowledge code)*; Representations *(knowledge code)* | Procedural, open-ended, representational; Symbolic, visual, verbal. |
